# Supplementary material for: Statistical Evidence for a Helical Nascent Chain
Source: Biomolecules. 2021 Feb 26;11(3):357. doi: 10.3390/biom11030357 (PMC7996779; doi:10.3390/biom11030357)
Supplement: Supplementary file 1 [file biomolecules-11-00357-s001.zip › Suppl-information-Figures-1-11.pdf]

In this file are the figures obtained with the large set of 444520 proteins, listed in the file `list-of-444520-proteins.dat`.

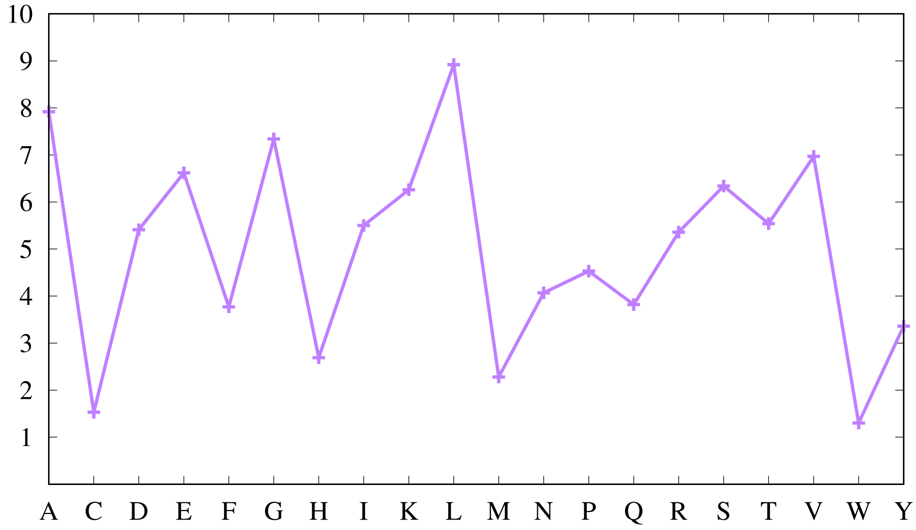


Figure 1: Average abundance, $\bar{f}_a(a)$ (cf. eq.(3) in the main text), of amino acid $a$, in the large protein set used. The values are given in percentage of the total number of amino acids (see main text). The large protein set was used.

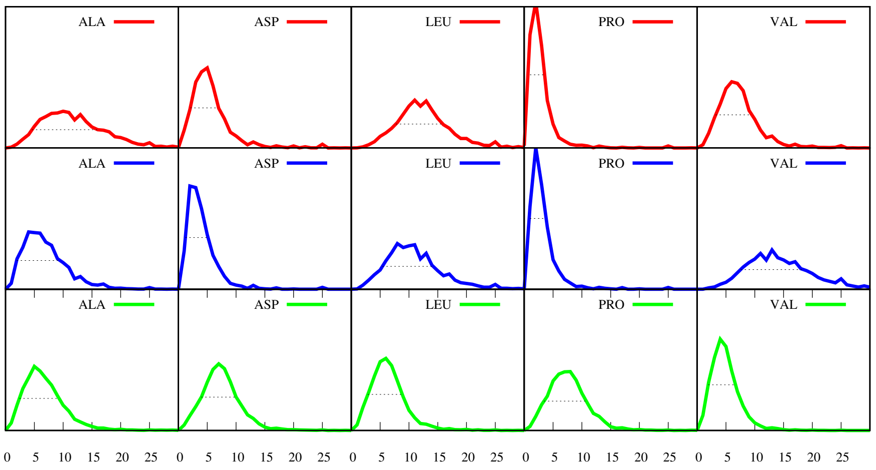


Figure 2: Distributions/histograms for a few of the frequencies $f(a,s,p)$, where the amino acid $a$ is specified at the top of each plot and where red is for $s = \alpha$-helix, blue is for $s = \beta$-sheets and green is for $s =$loops. In this figure, the variable $f(a,s,p)$ (the x-coordinate) runs from zero (which means that none of the amino acids $a$ are found in $s$) to 30 (which means that 30% of the amino acids in the protein are $a$'s found in $s$). The y-coordinate is proportional to the number of proteins with a given value of $f(a,s,p)$. The scale of the y-coordinate is the same for all plots and all distributions are normalised. The horizontal dotted lines are the FWHM of the distributions (see main text). The large protein set was used.

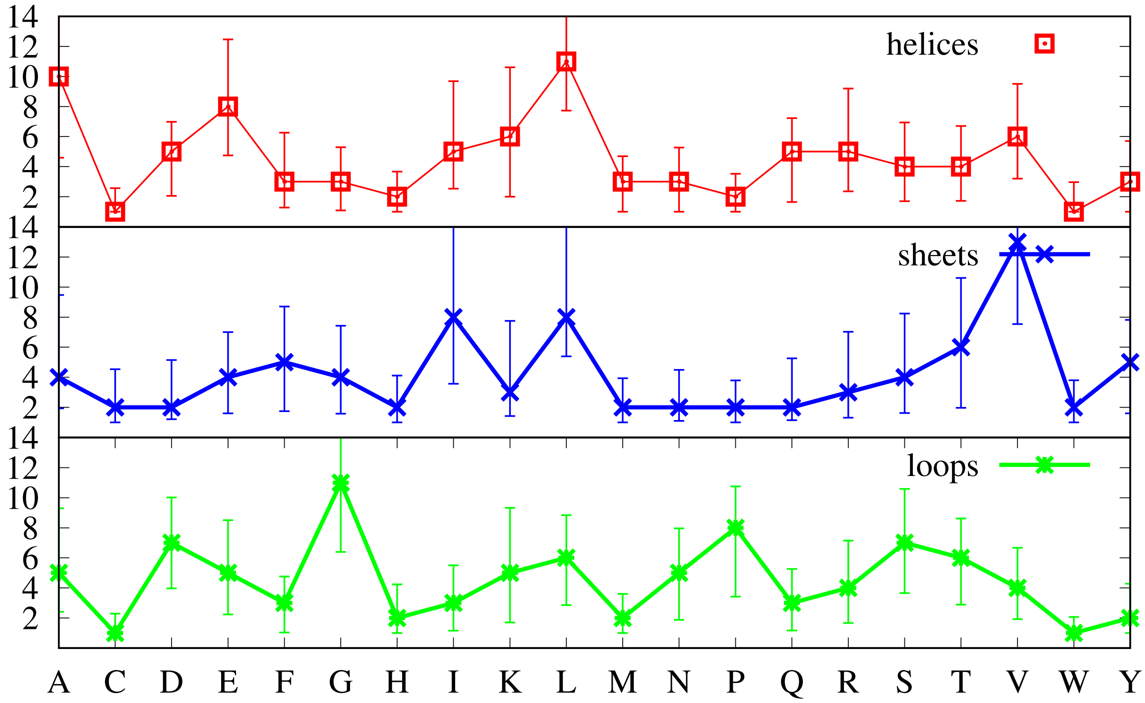


Figure 3: Average frequency of finding an amino acid $a$ in $\alpha$-helices (top plot), $\beta$-strands (middle plot) and loops (bottom plot). The values are given in percentage for each secondary structure, i.e. summing all the values *in each line* leads to 100. The amino acids are specified by their one letter codes. The large protein set was used.

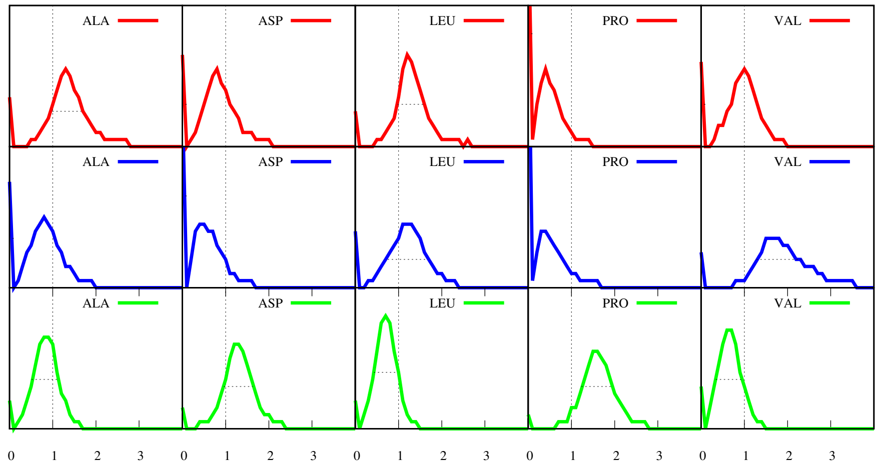


Figure 4: Distributions/histograms for a few of the ratios $R(a,s,p)$ (cf. Eq.(10) in the main text), where the amino acid $a$ is specified at the top of each plot and where red is for $s = \alpha$-helix, blue is for $s = \beta$-sheets and green is for $s =$loops. The variable $R(a,s,p)$ (the x-coordinate) runs from zero to four, the scale of the ordinates is the same in all plots and all histograms are normalised. The vertical dotted line marks the value $R(a,s,p) = 1$, when the actual number of amino acids $a$ in secondary structure $s$ is equal to what is expected in the absence of any correlation between $a$ and $s$. The horizontal dotted lines are the FWHM of the distributions (see main text). The large protein set was used.

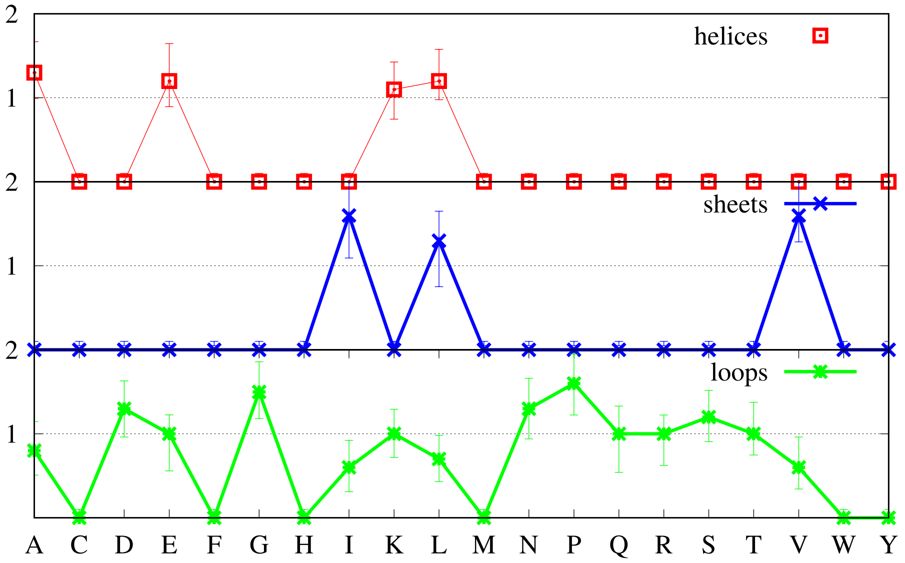


Figure 5: Most probable values for the ratios $R(a, s, p)$ (cf. Eq.(10) in the main text) for $\alpha$-helices (top plot, red), $\beta$-sheets (middle plot, blue) and loops (bottom plot, green). The most probable value is taken from the full distributions for each ratio $R(a, s, p)$ and the uncertainty around that value is given by the FWHM. The large protein set was used.

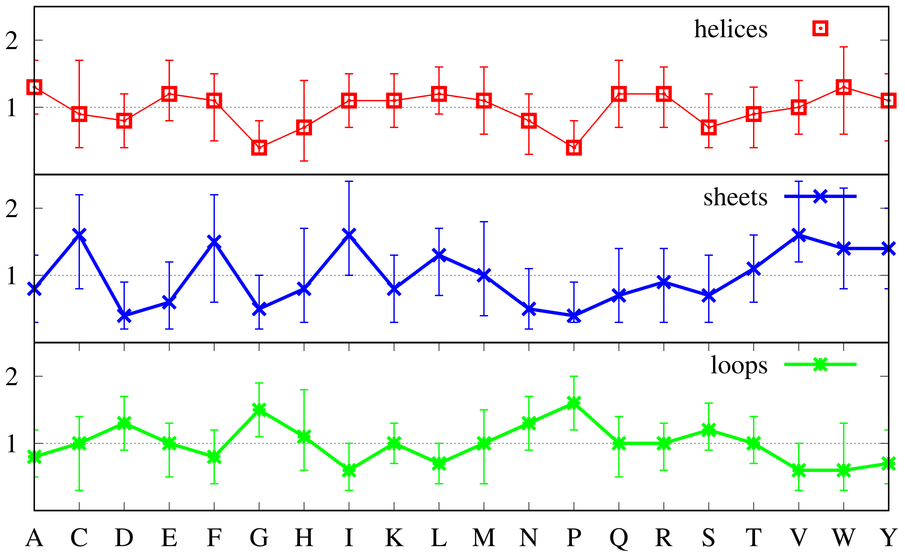


Figure 6: Most probable values for the ratios $R(a, s, p)$ (cf. Eq.(10) in the main text) for $\alpha$-helices (top plot, red), $\beta$-sheets (middle plot, blue) and loops (bottom plot, green). The most probable value is taken from the middle peak in the distributions for each ratio $R(a, s, p)$ and the uncertainty around that value is given by the FWHM of that middle peak. The large protein set was used.

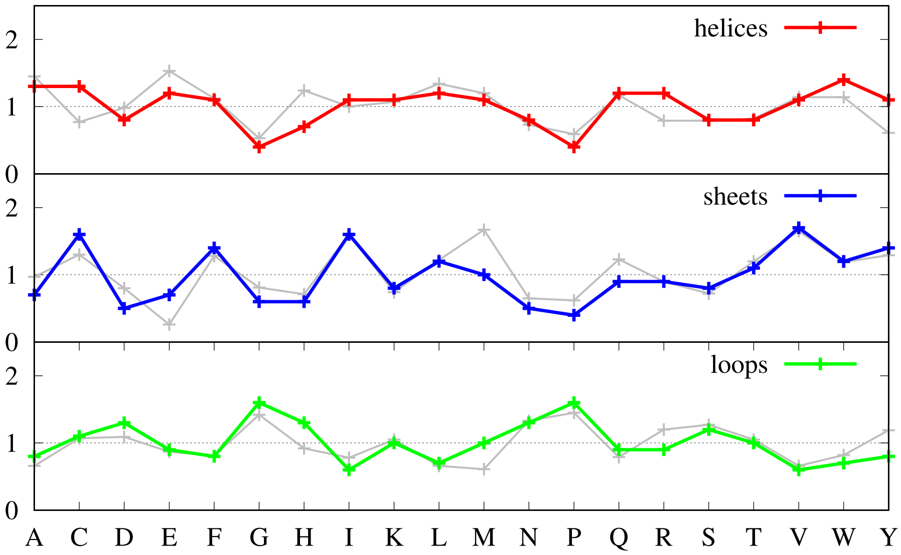


Figure 7: Most probable values for the ratios $R(a, s, p)$ (cf. Eq.(10) in the main text) for $\alpha$-helices (top plot, red), $\beta$-sheets (middle plot, blue) and loops (bottom plot, green). The most probable value is taken from the middle peak in the distributions for each ratio $R(a, s, p)$, as figure 6 of main text. In grey are the corresponding values calculated by Chou and Fasman in [1].

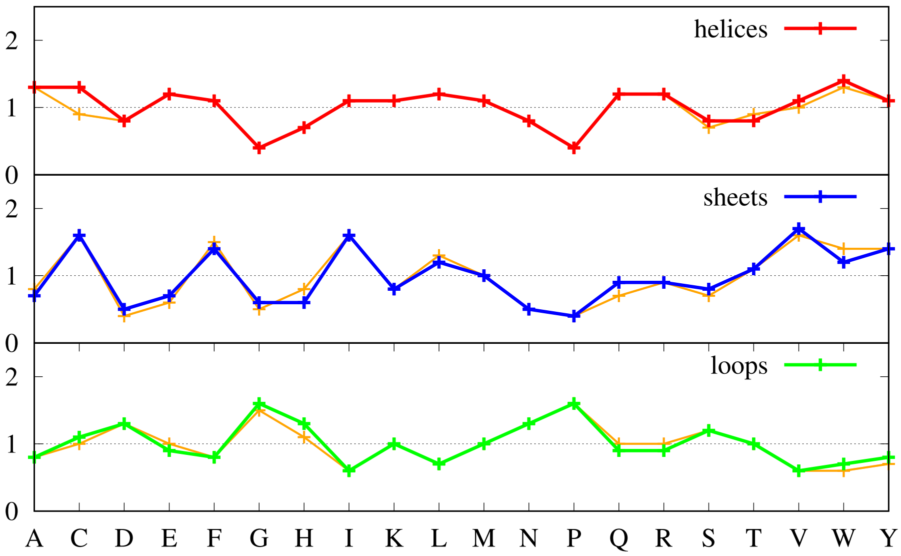


Figure 8: Most probable values for the ratios $R(a, s, p)$ (cf. Eq.(10) in the main text) for $\alpha$-helices (top plot, red), $\beta$-sheets (middle plot, blue) and loops (bottom plot, green). The most probable value is taken from the middle peak in the distributions for each ratio $R(a, s, p)$, as in figure 6 in main text. In orange are the corresponding values using the large protein set, as in figure 6 in this file.

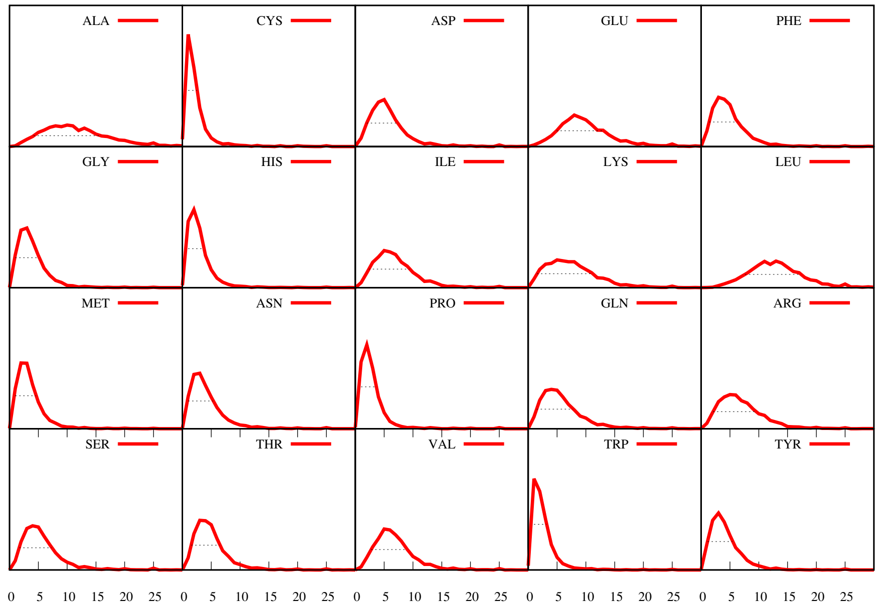


Figure 9: Distributions/histograms for a few of the frequencies $f(a, s, p)$, for $s = \alpha$-helix. The amino acid $a$ is specified at the top of each plot. In this figure, the variable $f(a, s, p)$ (the x-coordinate) runs from zero (which means that none of the amino acids $a$ are found in $s$) to 30 (which means that 30% of the amino acids in the protein are $a$'s found in $s$). The y-coordinate is proportional to the number of proteins with a given value of $f(a, s, p)$. The scale of the y-coordinate is the same for all plots and all distributions are normalised. The horizontal dotted lines are the FWHM of the distributions (see main text). The set of 13413 proteins listed in the file `list-of-13413-proteins.dat` was used.

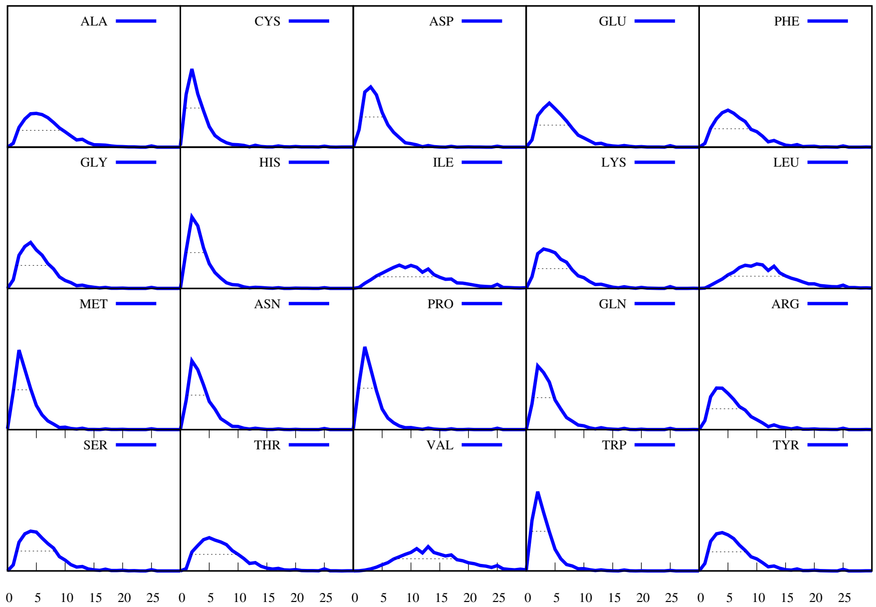


Figure 10: Distributions/histograms for a few of the frequencies $f(a, s, p)$, for $s = \beta$-sheet. The amino acid $a$ is specified at the top of each plot. In this figure, the variable $f(a, s, p)$ (the x-coordinate) runs from zero (which means that none of the amino acids $a$ are found in $s$) to 30 (which means that 30% of the amino acids in the protein are $a$'s found in $s$). The y-coordinate is proportional to the number of proteins with a given value of $f(a, s, p)$. The scale of the y-coordinate is the same for all plots and all distributions are normalised. The horizontal dotted lines are the FWHM of the distributions (see main text). The set of 13413 proteins listed in the file `list-of-13413-proteins.dat` was used.

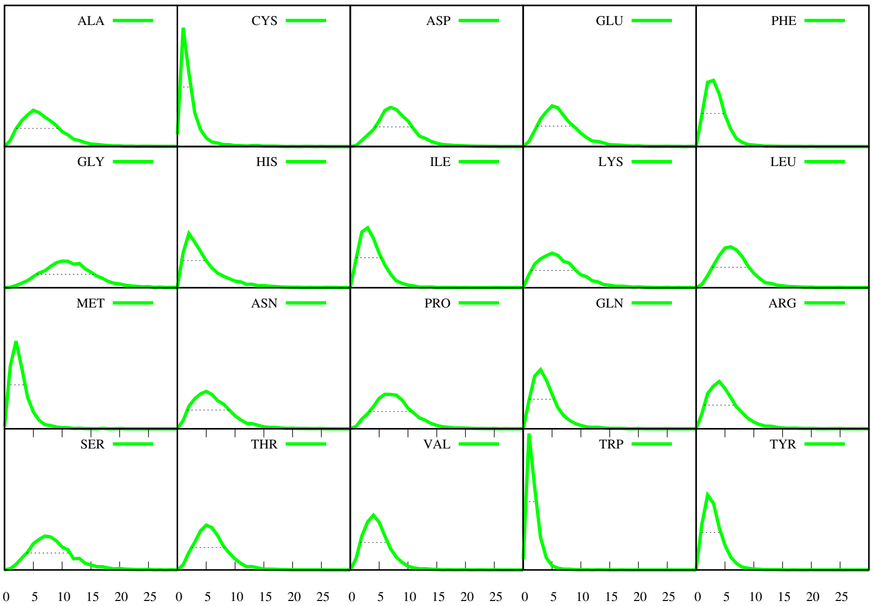


Figure 11: Distributions/histograms for a few of the frequencies $f(a, s, p)$, for $s$ =loop. The amino acid $a$ is specified at the top of each plot. In this figure, the variable $f(a, s, p)$ (the x-coordinate) runs from zero (which means that none of the amino acids $a$ are found in $s$) to 30 (which means that 30% of the amino acids in the protein are $a$'s found in $s$). The y-coordinate is proportional to the number of proteins with a given value of $f(a, s, p)$. The scale of the y-coordinate is the same for all plots and all distributions are normalised. The horizontal dotted lines are the FWHM of the distributions (see main text). The set of 13413 proteins listed in the file `list-of-13413-proteins.dat` was used.
